# Enhanced Oxidative Stress Is Responsible for TRPV4-Induced Neurotoxicity

**DOI:** 10.3389/fncel.2016.00232

**Published:** 2016-10-17

**Authors:** Zhiwen Hong, Yujing Tian, Yibiao Yuan, Mengwen Qi, Yingchun Li, Yimei Du, Lei Chen, Ling Chen

**Affiliations:** ^1^Department of Physiology, Nanjing Medical UniversityNanjing, China; ^2^The Laboratory Center for Basic Medical Sciences, Nanjing Medical UniversityNanjing, China; ^3^Research Center of Ion Channelopathy, Institute of Cardiology, Union Hospital, Tongji Medical College, Huazhong University of Science and TechnologyWuhan, China

**Keywords:** TRPV4, Ca^2+^, oxidative stress, NOS, neurotoxicity

## Abstract

Transient receptor potential vanilloid 4 (TRPV4) has been reported to be responsible for neuronal injury in pathological conditions. Excessive oxidative stress can lead to neuronal damage, and activation of TRPV4 increases the production of reactive oxygen species (ROS) and nitric oxide (NO) in many types of cells. The present study explored whether TRPV4-induced neuronal injury is mediated through enhancing oxidative stress. We found that intracerebroventricular injection of the TRPV4 agonist GSK1016790A increased the content of methane dicarboxylic aldehyde (MDA) and NO in the hippocampus, which was blocked by administration of the TRPV4 specific antagonist HC-067047. The activities of catalase (CAT) and glutathione peroxidase (GSH-Px) were decreased by GSK1016790A, whereas the activity of superoxide dismutase (SOD) remained unchanged. Moreover, the protein level and activity of neuronal nitric oxide synthase (nNOS) were increased by GSK1016790A, and the GSK1016790A-induced increase in NO content was blocked by an nNOS specific antagonist ARL-17477. The GSK1016790A-induced modulations of CAT, GSH-Px and nNOS activities and the protein level of nNOS were significantly inhibited by HC-067047. Finally, GSK1016790A-induced neuronal death and apoptosis in the hippocampal CA1 area were markedly attenuated by administration of a ROS scavenger Trolox or ARL-17477. We conclude that activation of TRPV4 enhances oxidative stress by inhibiting CAT and GSH-Px and increasing nNOS, which is responsible, at least in part, for TRPV4-induced neurotoxicity.

## Introduction

Oxidative stress is a state of imbalance between the level of the antioxidant defense mechanisms and the production of reactive oxygen species (ROS) and reactive nitrogen species (RNS; Simonian and Coyle, 1996). ROS mainly include superoxide anions, hydroxyl radicals and hydrogen peroxide (H_2_O_2_), and the major RNS include nitric oxide (NO), nitrogen dioxide and peroxynitrite (Bhat et al., 2015). Enzymatic and nonenzymatic antioxidants are cellular defense mechanisms that reduce the steady-state concentrations of ROS and RNS and repair oxidative cellular damage (Simonian and Coyle, 1996). Overproduction of free radicals can lead to necrotic cell damage and mediates apoptosis induced by a variety of stimuli (Loh et al., 2006). Growing evidence shows that oxidative stress is involved in mediating neuronal injury in diseases such as cerebral ischemia, Alzheimer’s disease (AD) and Parkinson’s disease (PD; Loh et al., 2006; Bhat et al., 2015). It has been shown that free radical production might be linked to a loss of cellular calcium (Ca^2+^) homeostasis and that Ca^2+^ overload is detrimental to mitochondrial function, leading to the generation of ROS in the mitochondria (Ermak and Davies, 2002). In the central nervous system (CNS), the expression of neuronal nitric oxide synthase (nNOS) accounts for the majority of NO activity, and increased intracellular Ca^2+^ levels can induce the production of NO through the stimulation of nNOS (Zhou and Zhu, 2009). Conversely, reciprocal interactions occur between Ca^2+^ and oxidative stress, which are involved in cellular damage (Ermak and Davies, 2002; Chinopoulos and Adam-Vizi, 2006; Kiselyov and Muallem, 2016).

The transient receptor potential (TRP) protein superfamily is a diverse group of Ca^2+^-permeable cation channels that are expressed in mammalian cells. Transient receptor potential vanilloid 4 (TRPV4) is a member of the TRP superfamily (Benemei et al., 2015). Activation of TRPV4 induces Ca^2+^ influx and increases the intracellular concentration of free Ca^2+^ ([Ca^2+^]_i_). Recent studies have reported that application of a TRPV4 agonist enhances the production of ROS in cultured human coronary artery endothelial cells and human coronary arterioles, which is dependent on TRPV4-mediated increases in [Ca^2+^]_i_ (Bubolz et al., 2012). Activation of TRPV4 elicits Ca^2+^ signal and stimulates H_2_O_2_ production in urothelial cells (Donkó et al., 2010). TRPV4 agonists significantly increase intracellular Ca^2+^ level and the production of superoxide in lung macrophages (Hamanaka et al., 2010). Ca^2+^ influx mediates the TRPV4-induced production of NO in the dorsal root ganglion following chronic compression and in the outer hair cells (Takeda-Nakazawa et al., 2007; Wang et al., 2015). These reports indicate that activation of TRPV4 may increase the production of ROS and RNS. TRPV4-induced toxicity has been confirmed in several types of cells, and activation of TRPV4 is responsible for neuronal injury in pathological conditions such as cerebral ischemic injury and AD (Li et al., 2013; Bai and Lipski, 2014; Jie et al., 2015, 2016). In our recent studies, intracerebroventricular injection of a TRPV4 agonist induced neuronal death in the hippocampus (Jie et al., 2015, 2016). In the present study, we investigated the effects of TRPV4 activation on oxidative stress in the hippocampus and further explored the involvement of this action in TRPV4-induced neuronal injury.

## Materials and Methods

### Animals

Male mice (9–10 weeks old, ICR, Oriental Bio Service Inc., Nanjing, China) were used. All experimental procedures conformed to the Guidelines for Laboratory Animal Research of Nanjing Medical University and were approved by the Institutional Animal Care and Use Committee of Nanjing Medical University.

### Drug Treatment

Drugs were intracerebroventricularly (icv.) injected as previously reported (Jie et al., 2016). Mice were anesthetized and placed in a stereotaxic device (Kopf Instruments, Tujunga, CA, USA). Drugs were injected into the right lateral ventricle (0.3 mm posterior, 1.0 mm lateral and 2.5 mm ventral to bregma) using a stepper-motorized micro-syringe (Stoelting, Wood Dale, IL, USA). GSK1016790A, HC-067047 and Trolox were first dissolved in DMSO and then in 0.9% saline to a final volume of 2 μl with a DMSO concentration of 1%. GSK1016790A is a specific agonist of TRPV4 and HC-067047 is a specific antagonist of it (Vincent and Duncton, 2011). GSK1016790A (0.1–5 μM/mouse) was injected once. HC-067047 was injected 30 min before the GSK1016790A injection and subsequently injected every 8 h for 3 days. ARL-17477 is a selective nNOS inhibitor and Trolox is a ROS scavenger (Zhang et al., 1996; Fang et al., 2013). ARL-17477 (1 μM/mouse) and Trolox (150 μM/mouse) were injected 30 min before the GSK1016790A injection and then injected once daily for 3 days. The doses of the drugs listed above were chosen according to previous reports (Zhang et al., 1996; Fang et al., 2013; Jie et al., 2016). Mice were randomly divided into the following groups: GSK1016790A group (mice were injected with GSK1016790A, GSK1016790A-injected mice), HC-067047 group (mice were injected with HC-067047), ARL-17477 group (mice were injected with ARL-17477), Trolox group (mice were injected with Trolox), GSK1016790A+HC-067047 group (mice were co-injected with GSK1016790A and HC-067047), GSK1016790A+ARL-17477 group (mice were co-injected with GSK1016790A and ARL-17477), GSK1016790A+Trolox group (mice were co-injected with GSK1016790A and Trolox) and control group (mice were injected with the same volume of the vehicle). In the following experiments, each group contained nine mice.

### Histological Examination

Histological examination of the hippocampal CA1 region was performed 3 days after the GSK1016790A injection. Mice were anesthetized and transcardially perfused with ice-cold phosphate-buffered saline (PBS) followed by 4% paraformaldehyde. After the brains were removed, they were placed in fixative at 4°C overnight and processed for paraffin embedding. Coronal sections (5 μm) were cut from the level of the hippocampus and used for toluidine blue or Hoechst staining. For toluidine blue staining, the pyramidal cells were identified using a conventional light microscope (Olympus DP70, Olympus, Tokyo, Japan) with a 40× objective. For Hoechst staining, the slices were stained with Hoechst-33342, and Hoechst-positive (Hoechst^+^) cells were counted using a fluorescence microscope (Olympus PD70, Olympus, Tokyo, Japan) with a 40× objective.

The neurons survived or Hoechst^+^ cells were counted in six sections per mouse, and the density was expressed as the number of cells per millimeter of length along the hippocampal CA1 pyramidal layer (Jie et al., 2015, 2016). The density of the surviving neurons or Hoechst^+^ cells in mice injected with GSK1016790A or GSK1016790A and Trolox/ARL-17477 was expressed as a percentage of the corresponding cells in the control mice.

### Western Blot Analysis

Western blot analysis was performed 3 days after the GSK1016790A injection as previously reported (Li et al., 2013; Jie et al., 2016). In brief, the hippocampi were collected and homogenized in a lysis buffer containing 50 mM Tris-HCl (pH 7.5), 150 mM NaCl, 5 mM EDTA, 10 mM NaF, 1 mM sodium orthovanadate, 1% Triton X-100, 0.5% sodium deoxycholate, 1 mM phenylmethylsulfonyl fluoride and a protease inhibitor cocktail (Complete; Roche, Mannheim, Germany). Protein concentrations were determined using a Bicinchoninic acid (BCA) Protein Assay Kit (Pierce, Rochford, IL, USA). Total proteins were separated using sodium dodecyl sulfate-polyacrylamide gel electrophoresis (SDS-PAGE) and were then transferred to a polyvinylidene difluoride (PVDF) membrane. The membranes were incubated with 5% nonfat dried milk in Tris-buffered saline containing 0.1% Tween 20 (TBST) for 60 min at room temperature and were then incubated with primary antibodies against nNOS (1:1000, Cell Signaling Technology, Boston, MA, USA) and glyceraldehyde-3-phosphate dehydrogenase (GAPDH; 1:5000, Abcam, Cambridge, MA, USA). Hippocampal samples were collected from the hemisphere of three mice as a set of western blot analysis. The summarized data represent the average of three experimental sets.

### nNOS Activity Assay

The activity of nNOS was measured 3 days after the GSK1016790A injection using a commercially available kit (Calbiochem, San Diego, CA, USA) according to previously reported methods (Zhu et al., 2016). The activity of nNOS was calculated by subtracting the activity of inducible NOS (iNOS) from the total NOS activity with the inhibited fraction of endothelial NOS (eNOS). The activity of nNOS in the GSK1016790A-injected mice was normalized to the activity in the control mice.

### Enzyme-Linked Immunosorbent Assay (ELISA)

Enzyme-Linked Immunosorbent Assays (ELISA) were performed 3 days after the GSK1016790A injection as previously reported (Li et al., 2010). The content of methane dicarboxylic aldehyde (MDA) and NO, and the activity of catalase (CAT), superoxide dismutase (SOD) and glutathione peroxidase (GSH-Px) in the hippocampal homogenates were determined using an ELISA according to the manufacturer’s instructions (Nanjing Jiancheng Biochemistry Co., Nanjing, China), and the content or activity in the mice injected with GSK1016790A and/or antagonist was normalized to the control values.

### Chemicals

All chemicals, unless otherwise stated, were obtained from Sigma Chemical Company.

### Data Analysis

Data are expressed as mean ± S.E.M and were analyzed using Stata 7.0 software (STATA Corporation, USA). The statistical analysis was conducted using analysis of variance (ANOVA) followed by Bonferroni’s *post hoc* test, and significance levels were set at *P* < 0.05 and *P* < 0.01. The dose-response curve was fit using the Hill equation in which *a* = *a*_max_/[1+(EC_50_/*C*)^*n*^], with *n* being the Hill coefficient and EC_50_ being the dose of GSK1016790A that produced a 50% effect.

## Results

### Effect of the TRPV4 Agonist GSK1016790A on the MDA and NO Content in the Hippocampus

**Figure 1:**
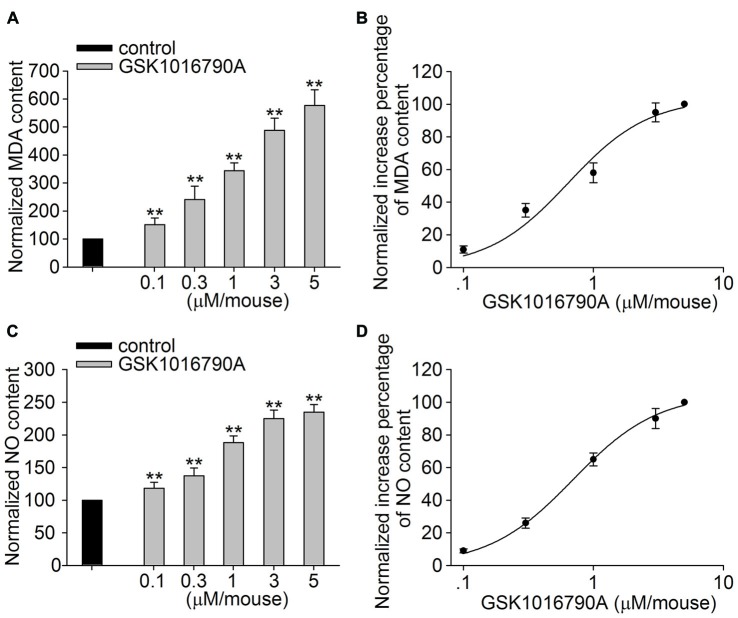
**Effect of the Transient receptor potential vanilloid 4 (TRPV4) agonist GSK1016790A on methane dicarboxylic aldehyde (MDA) and nitric oxide (NO) content. (A,B)** The MDA content in the hippocampus was increased in a dose-dependent manner by the application of the TRPV4 agonist GSK1016790A (0.1–5 μM/mouse), and the EC_50_ and *n* values of the dose-response curve were 0.63 ± 0.14 μM/mouse and 1.40, respectively. **(C,D)** The NO content in the hippocampus was increased by injection of GSK1016790A (0.1–5 μM/mouse) in a dose-dependent manner, and the EC_50_ and *n* values of the dose-response curve were 0.69 ± 0.04 μM/mouse and 1.33, respectively. **P < 0.01 vs. control.

In this study, GSK1016790A was used to explore the effect of TRPV4 activation on oxidative stress in the hippocampus. Lipid peroxidation in the hippocampus was assessed by detecting the MDA content. As shown in Figure 1A, the MDA content was significantly increased in the GSK1016790A-injected mice compared to the control mice (*P* < 0.01). The GSK1016790A-induced increase in the MDA content was dose-dependent at doses ranging from 0.1 μM/mouse to 5 μM/mouse, and the EC_50_ value was 0.63 ± 0.14 μM/mouse (Figure 1B). Meanwhile, the NO content was higher in the GSK1016790A-injected mice than that in the control mice (*P* < 0.01; Figure 1C). As shown in Figure 1D, the GSK1016790A-induced increase in the NO content was also dose-dependent, and the EC_50_ value was 0.69 ± 0.04 μM/mouse at doses of GSK1016790A ranging from 0.1 μM/mouse to 5 μM/mouse. These results indicate that application of a TRPV4 agonist may increase the generation of ROS and NO. Because GSK1016790A at the dose of 1 μM/mouse caused a significant increase in the MDA (343.77 ± 27.79%) and NO content (188.17 ± 10.10%), and this dose of GSK1016790A has been shown to induce neurotoxicity in previous studies (Jie et al., 2015, 2016), GSK1016790A at the dose of 1 μM/mouse was used in the following experiments.

### Effect of GSK1016790A on the Activity of Antioxidant Enzymes and the Protein Level and Activity of nNOS in the Hippocampus

**Figure 2:**
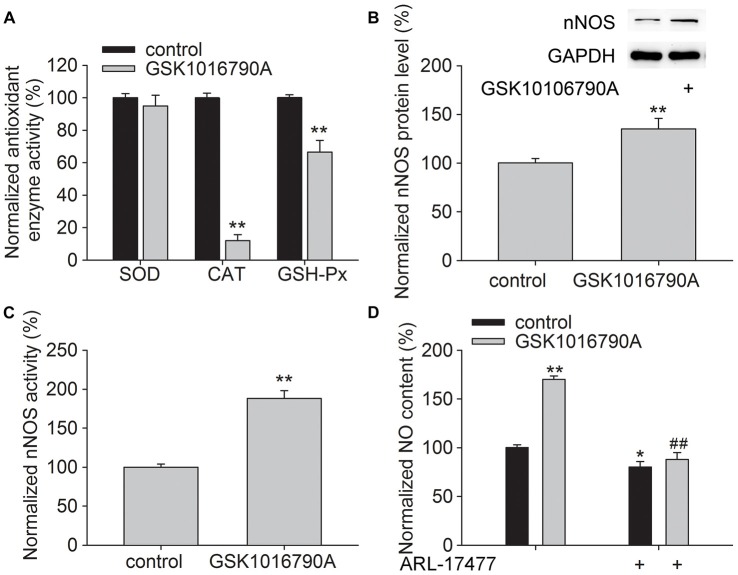
**Effect of GSK1016790A on the antioxidant enzyme activity and neuronal nitric oxide synthase (nNOS) protein level and activity. (A)** The activities of catalase (CAT) and glutathione peroxidase (GSH-Px) were reduced by GSK1016790A, whereas superoxide dismutase (SOD) activity was unaffected. **(B,C)** The protein level **(B)** and activity **(C)** of nNOS were increased by GSK1016790A. **(D)** The NO content in the hippocampus was increased by injection of GSK1016790A, which was significantly blocked by the nNOS inhibitor ARL-17477. *P < 0.05 and **P < 0.01 vs. control, ^##^*P* < 0.01 vs. GSK1016790A.

Antioxidant enzymes responsible for endogenous defense include GSH-Px, CAT and SOD. As shown in Figure 2A, the activities of CAT and GSH-Px were significantly lower in the GSK1016790A-injected mice (CAT: 12.07 ± 3.62%, GSH-Px: 66.55 ± 7.15%, *P* < 0.01), whereas the activity of SOD was unchanged, which implies that CAT and GSH-Px are selectively inhibited by the TRPV4 agonist.

In addition, the protein level and activity of nNOS were significantly increased in the GSK1016790A-injected mice (Figures 2B,C; *P* < 0.01). We also found that the increased NO content in the GSK1016790A-injected mice was attenuated by application of ARL-17477 which is an nNOS specific inhibitor (Figure 2D; *P* < 0.01). These results indicate that application of a TRPV4 agonist enhances NO production, which is likely mediated through increasing nNOS activity and expression.

### Effect of Administration of a TRPV4 Antagonist on the GSK1016790A-Induced Changes in the MDA and NO Content, Activity of Antioxidant Enzymes and Expression and Activity of nNOS

**Figure 3:**
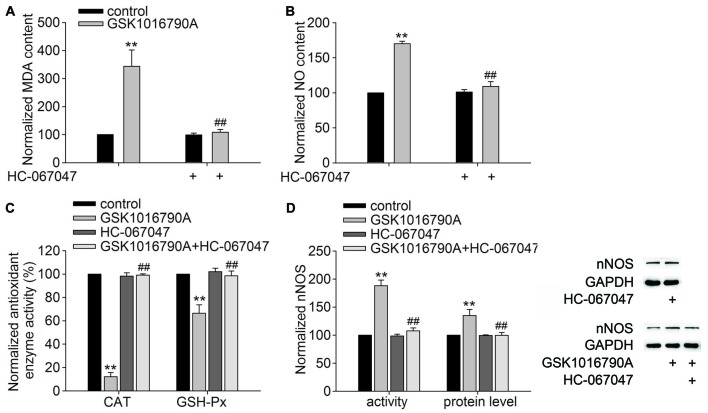
**Effect of the TRPV4 specific antagonist HC-067047 on the GSK1016790A-induced modulations of the MDA and NO content, CAT and GSH-Px activities and nNOS protein level and activity. (A,B)** HC-067047 had no effect on the MDA or NO content in the control mice, but significantly blocked the increase in MDA **(A)** and NO **(B)** content in the GSK1016790A-injected mice. **(C)** The GSK1016790A-induced decrease in CAT and GSH-Px activities was significantly blocked by HC-067047. **(D)** The increase in both the protein level and activity of nNOS in the GSK1016790A-injected mice was significantly attenuated by HC-067047. **P < 0.01 vs. control, ^##^*P* < 0.01 vs. GSK1016790A.

The TRPV4 specific antagonist HC-067047 (10 μM/mouse) was used to further confirm the role of TRPV4 in the above GSK1016790A-induced actions. As shown in Figures 3A,B, the MDA and NO content was significantly decreased in the mice co-injected with GSK1016790A and HC-067047 (MDA: 108.45 ± 10.41%, NO: 109.00 ± 7.01%) compared to GSK1016790A-injected mice (*P* < 0.01). Figure 3C shows that the activities of CAT and GSH-Px in the mice co-injected with GSK1016790A and HC-067047 were significantly increased (CAT: 99.07 ± 1.14%, GSH-Px: 98.55 ± 4.15%) compared to GSK1016790A-injected mice (*P* < 0.01). Moreover, the increase in both the protein level and activity of nNOS in the GSK1016790A-injected mice were significantly blocked by HC-067047 (*P* < 0.01; Figure 3D). Collectively, the present results indicate that activation of TRPV4 may enhance oxidative stress, which may be mediated through inhibiting CAT and GSH-Px, and enhancing nNOS expression and activity.

### Effect of Administration of a ROS Scavenger and an nNOS Inhibitor on TRPV4 Agonist-Induced Neuronal Injury

**Figure 4:**
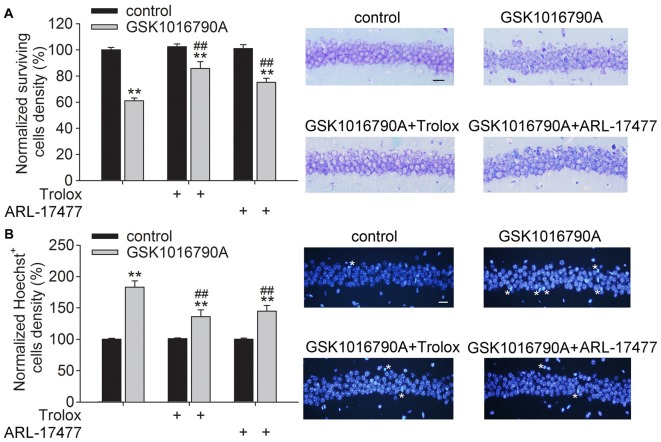
**Effect of a reactive oxygen species (ROS) scavenger and an nNOS inhibitor on GSK1016790A-induced neuronal injury. (A)** Injection of the TRPV4 agonist GSK1016790A reduced the number of surviving cells in the hippocampal CA1 area, which was significantly attenuated by the ROS scavenger Trolox and the nNOS inhibitor ARL-17477. Scale bar = 50 μM. **(B)** More Hoechst^+^ cells (white stars) were found in the hippocampal CA1 area in the GSK1016790A-injected mice, which was markedly blocked by Trolox and ARL-17477. Scale bar = 50 μM. **P < 0.01 vs. control, ^##^*P* < 0.01 vs. GSK1016790A.

In the present study, the densities of the surviving neurons and Hoechst^+^ cells in the control group were 327 ± 15.64/mm and 4.14 ± 1.93/mm, respectively, which were consistent with previous reports (Jie et al., 2015, 2016). Activation of TRPV4 has been shown to induce toxicity. As shown in Figure 4A, the number of surviving cells was decreased by GSK1016790A treatment (GSK1016790A: 61.07 ± 2.03%), and more surviving neurons were found in the GSK1016790A-injected mice when they were co-injected with the ROS scavenger Trolox (GSK1016790A+Trolox: 85.78 ± 5.18%) or with ARL-17477 (GSK1016790A+ARL-17477: 75.13 ± 3.14%; *P* < 0.01). Additionally, more Hoechst^+^ cells were found in the GSK1016790-injected mice (183.07 ± 10.03%), which was significantly attenuated by administration of Trolox (135.90 ± 11.052%) or ARL-17477 (145.01 ± 9.14%; *P* < 0.01; Figure 4B). These results indicate that enhanced oxidative stress is responsible, at least in part, for the TPPV4 activation-induced neurotoxicity.

## Discussion

TRPV4 is widely expressed in the nervous system and is normally assembled as a homotetramer. TRPV4 can also be assembled as a heterotetramer (e.g., with TRPC1 in vascular endothelial cells, TRPP2 in tsA 201 cells and a heteromeric TRPV4-TRPC1-TRPP2 complex in primary cultured rat mesenteric artery endothelial cells and cotransfected HEK293 cells; Stewart et al., 2010; Ma et al., 2011; Du et al., 2014). However, it is still unknown whether these heterotetramers exist in the CNS. TRPV4 is sensitive to multiple stimuli including physical factors (e.g., hypotonicity-induced cell swelling, mild heat and mechanical stimulation), endogenous stimuli (e.g., arachidonic acid (AA) and its metabolites including epoxyeicosatrienoic acids) and synthetic chemicals (e.g., GSK1016790A and 4α-PDD; Vincent and Duncton, 2011). Increasing evidence suggests that activation of TRPV4 is involved in the pathogenesis of some nervous system diseases and is responsible for neuronal injury. For example, TRPV4 protein levels are up-regulated during cerebral ischemia, and inhibition of TRPV4 reduces brain infarction (Li et al., 2013; Jie et al., 2016). TRPV4 immunoreactivity is significantly increased in the cerebral cortex, hippocampal formation, striatum and thalamus in a mouse model of AD (Lee and Choe, 2016). β-amyloid peptide-1–40 (Aβ_1–40_) can activate astrocytic TRPV4 in the hippocampus, and TRPV4 antagonists reduce neuronal and astrocytic damage caused by Aβ_1–40_ (Bai and Lipski, 2014). Because TRPV4 is permeable to Ca^2+^, its activation induces Ca^2+^ influx (Benemei et al., 2015). Therefore, TRPV4-induced elevations in [Ca^2+^]_i_ have attracted significant attention in research aimed at exploring the mechanisms underlying TRPV4-mediated neuronal injury.

Oxidative stress refers to the cytopathological consequences of a mismatch between the production and elimination of free radicals and has been confirmed to be responsible for neuronal injury in pathological conditions (Simonian and Coyle, 1996; Loh et al., 2006; Bhat et al., 2015). Increased [Ca^2+^]_i_ can initiate a number of deleterious processes including activation of NOS and free radical generation (Ermak and Davies, 2002). Recent studies have reported that activation of TRPV4 enhances the production of ROS or NO in endothelial cells, urothelial cells, macrophages and outer hair cells, which is related to TRPV4-mediated Ca^2+^ signaling (Takeda-Nakazawa et al., 2007; Donkó et al., 2010; Hamanaka et al., 2010; Bubolz et al., 2012; Wang et al., 2015). Consistent with these results, the present study showed that application of the TRPV4 agonist GSK1016790A increased the MDA and NO content in the hippocampus (Figure 1). It has been reported that activation of N-Methyl-D-Aspartate (NMDA) glutamate receptors results in increased nNOS-mediated NO generation (Yamada and Nabeshima, 1997). In the hippocampus, activation of TRPV4 enhances NMDA receptor-mediated Ca^2+^ influx (Li et al., 2013), which may contribute to TRPV4-induced increases in [Ca^2+^]_i_ and the production of free radicals. NO is derived from three isoforms of NOS (nNOS, eNOS and iNOS), of which nNOS and iNOS have been reported to be involved in neuronal injury during the early and late stages of cerebral ischemia, respectively (Zhang et al., 1996; ArunaDevi et al., 2010). In this study, we found that the protein level and activity of nNOS were increased by treatment with GSK1016790A (Figures 2B,C), and an nNOS specific inhibitor ARL-17477 blocked the GSK1016790A-induced increase in NO content (Figure 2D), which indicated that application of the TRPV4 agonist may enhance nNOS resulting in increased NO production. The present study also showed that the activities of CAT and GSH-Px were selectively reduced by GSK1016790A (Figure 2A). It was also noted that the GSK1016790A-induced increase in MDA and NO content was significantly blocked by the TRPV4 specific antagonist HC-067047. In addition, the GSK1016790A-induced increase in nNOS protein level and activity and the inhibition of CAT and GSH-Px activities were significantly blocked by HC-067047 (Figure 3). These results further confirmed that activation of TRPV4 might enhance oxidative stress in the hippocampus, which may be a result of increased free radicals production and decreased elimination of free radicals.

It has been shown that inhibition of TRPV4 increases the viability of astrocytes following an oxidative stress insult (Bai and Lipski, 2010). TRPV4 contributes to Aβ_1–40_-induced neuronal and astrocytic damage, which is related to oxidative stress (Bai and Lipski, 2014). We recently reported TRPV4-induced neurotoxicity *in vivo* (Jie et al., 2015, 2016). Here, GSK1016790A-induced neuronal loss and apoptosis in the hippocampal CA1 area were significantly blocked by a ROS scavenger and an nNOS specific inhibitor, which indicated that the TRPV4-induced neurotoxicity was mediated, at least in part, through enhanced oxidative stress (Figure 4). Our unpublished data show that inhibition of TRPV4 reduces infarction in a myocardial ischemia-reperfusion model through inhibition of ROS production (Du and Chen, unpublished data). Although different mechanisms underlie cerebral and myocardial ischemia, excessive oxidative stress plays an important role in both neuronal and myocardial injury. Therefore, it is proposed that TRPV4-mediated enhancement of oxidative stress is likely responsible for the neuronal injury in cerebral ischemia injury; however, this hypothesis should be further confirmed.

The present study showed that enhanced oxidative stress was involved in TRPV4-induced hippocampal neuronal injury. Conversely, some TRP channels can potentially act as sensors of changes in the cellular redox status and contribute to ROS-induced increases in intracellular Ca^2+^ concentrations (Badr et al., 2016; Ogawa et al., 2016). It has been reported that TRPV4 can be activated by NO and H_2_O_2_ (Yoshida et al., 2006; Badr et al., 2016). Therefore, it is possible that TRPV4 and free radicals may form a positive feedback loop that is involved in neuronal injury under pathological conditions. Combined with previous reports, this study indicates that targeting TRPV4 might be a potential strategy for neuronal protection.

## Author Contributions

Lei Chen conceived and designed the study. ZH, YT and YY performed the experiments. YL, MQ and YD performed data collection and analysis. Ling Chen revised the manuscript. Lei Chen prepared the manuscript and is responsible for publication decisions.

## Conflict of Interest Statement

The authors declare that the research was conducted in the absence of any commercial or financial relationships that could be construed as a potential conflict of interest.
